# Configuration Analysis of the ERS Points in Large-Volume Metrology System

**DOI:** 10.3390/s150924397

**Published:** 2015-09-22

**Authors:** Zhangjun Jin, Cijun Yu, Jiangxiong Li, Yinglin Ke

**Affiliations:** The State Key Laboratory of Fluid Power Transmission and Control, Zhejiang University, Hangzhou 310058, China; E-Mails: leopard85@zju.edu.cn (Z.J.); ljxiong@zju.edu.cn (J.L.); ylke@zju.edu.cn (Y.K.)

**Keywords:** aircraft assembly, large-volume metrology, transformation matrix errors, configuration of the ERS points, layout of the ERS points

## Abstract

In aircraft assembly, multiple laser trackers are used simultaneously to measure large-scale aircraft components. To combine the independent measurements, the transformation matrices between the laser trackers’ coordinate systems and the assembly coordinate system are calculated, by measuring the enhanced referring system (ERS) points. This article aims to understand the influence of the configuration of the ERS points that affect the transformation matrix errors, and then optimize the deployment of the ERS points to reduce the transformation matrix errors. To optimize the deployment of the ERS points, an explicit model is derived to estimate the transformation matrix errors. The estimation model is verified by the experiment implemented in the factory floor. Based on the proposed model, a group of sensitivity coefficients are derived to evaluate the quality of the configuration of the ERS points, and then several typical configurations of the ERS points are analyzed in detail with the sensitivity coefficients. Finally general guidance is established to instruct the deployment of the ERS points in the aspects of the layout, the volume size and the number of the ERS points, as well as the position and orientation of the assembly coordinate system.

## 1. Introduction

Large-volume metrology system has become central throughout the aircraft assembly process [1]. Before the aligning and joining of the aircraft components, the positions and orientations of the components should be measured [2]. To overcome the obstacle of the large aircraft components or the jigs, multiple laser trackers are used simultaneously to measure large-scale aircraft components. To combine the measurement results from multiple laser trackers into the assembly coordinate system, the rigid body registration [3] process is implemented to calculate the optimal transformation matrices (rotation matrix R, translateion matrix T) between the laser tracker’s coordinate system and the assembly coordinate system, by measuring the coordinates of the enhanced reference system (ERS) points. The ERS points constitute the reference of the assembly coordinate system, which is properly deployed to envelope the entire assembly volume. The configuration of the ERS points is crucial to the uncertainties of the transformation matrix errors, which mostly determine the measurement uncertainty of the large-volume metrology system. However, the ERS points have been usually deployed experientially so far, and there is little guidance that can be followed to instruct the deployment of the ERS points.

The rigid-body registration problem is usually resolved with the singular value decomposition (SVD) method [4,5], unit quaternion method [6], orthonormal matrix method [7] or the iterative closest point (ICP) method [8]. To improve the measurement accuracy, the Boeing Company [9] used the dynamic weighting method for the bundle adjustment to reduce the registration error (RE). Calkins [10,11] described a unified spatial metrology network to properly combine the nominal data and the measurement data from multiple types of instruments and thereafter use uncertainty fields to evaluate the measurement accuracy. Predmore [12] proposed a mahalanobis bundle adjustment method to determine the best-fitted transformation matrices, which fully took into account the uncertainty ellipsoid of each measured point. Mitchell [13] used the geometric fusion method to reduce the data combination error from multiple laser trackers. However, these studies only paid attention to the reduction of the RE; there is still deficiency in the estimation of the uncertainties of the transformation matrix errors, and little attention has been paid to the influence of the configuration of the ERS points that affect the transformation matrix errors.

To optimize the deployment of the ERS points, an explicit model is derived to estimate the uncertainties of the transformation matrix errors in the registration process, using the configuration matrix of the ERS points. Based on the derived model, a group of sensitivity coefficients are proposed to evaluate the quality of the configuration of the ERS points. Then, the sensitivity coefficients of several typical configurations of the ERS points are analyzed in details. According to the analysis result, general guidance is established for the deployment of the ERS points in the aspects of the layout, the volume size, and the number of the ERS points, as well as the position and orientation of the assembly coordinate system.

## 2. Measurement Uncertainty Estimation Model

**Figure 1:**
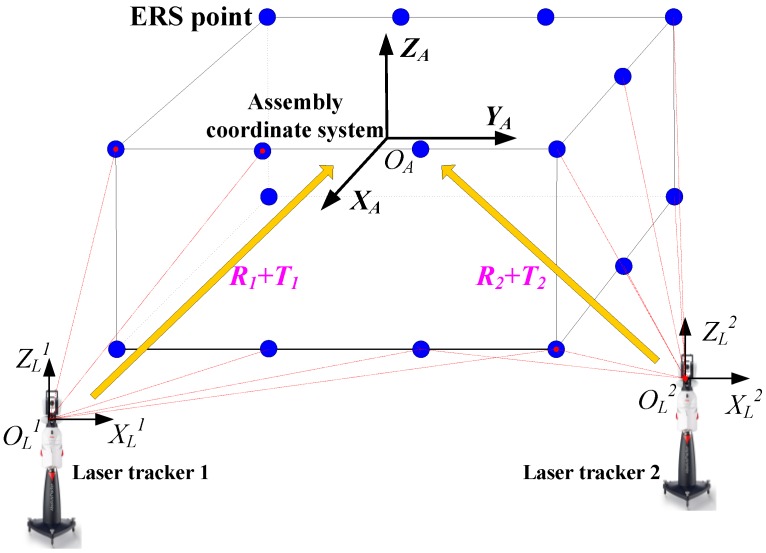
The measurement principle in large-scale aircraft assembly systems.

In a large-scale aircraft assembly process, multiple laser trackers are needed to work cooperatively to acquire the requisite number of measurements. The measurement data from different laser trackers are unrelated and should be transformed into the assembly coordinate system to evaluate the aircraft alignment accuracy. As the working locations of laser trackers are not pre-defined; the actual transformation matrices (R, T) from the laser tracker’s coordinate system to the assembly coordinate system are unknown. The rigid body registration process is implemented to obtain the optimal transformation matrices, by measuring the coordinates of the ERS points. The ERS points constitute the reference of the assembly coordinate system and they are deployed dispersedly to cover the assembly volume (Figure 1).

After the registration process, the measurements from the laser trackers (***P_L_***) are transformed into the assembly coordinate system (***P_A_***) using the following formula:

(1) $$P_{A}=RP_{L}+T$$

Obviously, the transformation matrix errors mostly determine the measurement precision. To understand measurement uncertainty, an error propagation model in the registration process is proposed, and the uncertainty evaluation models of the transformation matrix errors are obtained in the next section.

### 2.1. Error Propagation Model in Registration

Rigid body registration is used to determine the relationship between two coordinate systems with two related point sets. The fundamental problem of registration is to find the optimal rotation matrix ***R*** and translation vector ***T*** that minimizes *RE*:

(2) $$RE=\frac{1}{N}\sum_{i=1}^{N}{\left|y_{i}-\left(Rx_{i}+T\right)\right|}^{2}$$

where *x_i_* is the coordinate of the ERS point measured by the laser tracker, *y_i_* is the nominal coordinate of the ERS point in the assembly coordinate system, and *N* is the number of the ERS points.

As the ERS points comprise the fixed reference, the nominal coordinates of the ERS points are pre-defined, and the RE can be rewritten in Equation (3):

(3) $$RE=\frac{1}{N}\sum_{i=1}^{N}{\left|R\left(x_{i}+\Delta x_{i}\right)+T-y_{i}\right|}^{2}$$

where *x_i_* is the nominal coordinate of the ERS point in the laser tracker’s coordinate system, and ∆*x_i_* represents the measurement error of the ERS point from the laser tracker, which is supposed to be normal distributed [14]: ∆*x_i_*~*N*(0,***U_xi_***), and ***U_xi_*** is a 3 × 3 diagonal uncertainty matrix.

Let us define *R*_0_ and *T*_0_ as the nominal transformation matrices, which exactly satisfy the following equation:

(4) $$y_{i}=R_{0}x_{i}+T_{0}$$

Then using the formula in Equation (4), we get the *RE* as follows:

(5) $$\begin{array}{cc} RE & =\frac{1}{N}\sum_{i=1}^{N}{\left|R\left(x_{i}+\Delta x_{i}\right)+T-\left(R_{0}x_{i}+T_{0}\right)\right|}^{2}\\ & =\frac{1}{N}\sum_{i=1}^{N}{\left|\left(\Delta R-I\right)R_{0}x_{i}+\left(T-T_{0}\right)+R\Delta x_{i}\right|}^{2}\\ & =\frac{1}{N}\sum_{i=1}^{N}{\left|\bar{\Delta R}X_{i}+\Delta T+R\Delta x_{i}\right|}^{2} \end{array}$$

where $\bar{\Delta R}=\Delta R-I=\left[\begin{array}{ccc} 0 & -\Delta \gamma & \Delta \beta \\ \Delta \gamma & 0 & -\Delta \alpha \\ -\Delta \beta & \Delta \alpha & 0 \end{array}\right]$, and $\Delta T=T-T_{0}={\left[\Delta {\text{t}}_{x},\Delta {\text{t}}_{y},\Delta {\text{t}}_{z}\right]}^{T}$, $X_{i}=R_{0}x_{i}=y_{i}-T_{0}$.

In which ***X_i_*** indicates the layout of the ERS point in the assembly coordinate system, and Δ*α*, Δ*β*, Δ*γ*, Δ*t_x_*, Δ*t_y_*, and Δ*t_z_* represent the transformation parameter errors, particularly Δ*α*, Δ*β*, and Δ*γ* are the rotation parameter errors, and Δ*t_x_*, Δ*t_y_*, and Δ*t_z_* are the translation parameter errors.

The minimization of Equation (5) is equivalent to finding the least-square solution to the following over-determined set of 3N linear equations with the six unknown variables: Δ*α*, Δ*β*, Δ*γ*, Δ*t*_x_, Δ*t_y_*, and Δ*t*_z_:

(6) $${\left(\bar{\Delta R}X_{i}\right)}_{j}+{\left(\Delta T\right)}_{j}={\left(-R\Delta x_{i}\right)}_{j},i=1,2,\ldots N,j=1,2,3$$

where the subscript *j* enumerates the components of the three-dimensional vectors.

By defining the 6 × 1 transformation parameter error vector ***q*** and the 3*N* × 1 measurement error vector of the ERS points ***e*** as follows:

(7) $$q={\left[\Delta \alpha ,\Delta \beta ,\Delta \gamma ,\Delta t_{x},\Delta t_{y},\Delta t_{z}\right]}^{T}$$

(8) $$e={\left[\begin{array}{ccccc} \cdots & {\left(-R\Delta x_{i}\right)}_{1}, & {\left(-R\Delta x_{i}\right)}_{2}, & {\left(-R\Delta x_{i}\right)}_{3}, & \cdots \end{array}\right]}^{T}$$

where ***e*** is also normal distributed: ***e***~N(0, ***U***(***e***)), and ***U***(***e***) is a 3*N* × 3*N* diagonal matrix, for which the diagonal element *U*(*e_j_*), *j* = 1,…,3*N*, are defined by the following formulas: *U*(*e*_2*i*−1_) = [***RU_xi_ R^t^***]_11_, *U*(*e*_2*i*_) = [***RU_xi_ R^t^***]_22_, and *U*(*e*_2*i*+1_) = [***RU_xi_ R^t^***]_33_, *i* = 1,…,*N*.

Then, Equation (6) can be rewritten in matrix form as follows:

(9) Cq=e

where the matrix ***C*** is called the configuration matrix of the ERS points, which is defined in Equation (10):

(10) $$C={\left[\begin{array}{cc} \begin{array}{c} Z_{1}\\ \cdots \\ Z_{i}\\ \cdots \\ Z_{N} \end{array} & \begin{array}{c} I_{3}\\ \cdots \\ I_{3}\\ \cdots \\ I_{3} \end{array} \end{array}\right]}_{3N\times 6}$$

in which $Z_{i}=\left[\begin{array}{ccc} 0 & X_{i3} & -X_{i2}\\ -X_{i3} & 0 & X_{i1}\\ X_{i2} & -X_{i1} & 0 \end{array}\right]$, *X_ij_* is the *j*th component of *X_i_*, and $I_{3}$ is the unit matrix.

Considering the singular-value decomposition of matrix ***C***:

(11) $$C=U\Lambda V^{t}$$

then the least-squares solution of Equation (9) is given as follows:

(12) $$q=C^{+}e$$

where ***C*^+^** = ***VΛ*^+^*U^t^***, and ***Λ*^+^** is a 6 × 3N matrix for which ***Λ***^+^*_ii_* = ***Λ***^−1^*_ii_*, *i* = 1,2…,6.

### 2.2. Uncertainty Estimation of the Transformation Parameter Errors

With the assumption that the measurement error of the ERS point is normal distributed, the transformation parameter errors are also normal distributed. According to the GUM [15], the uncertainties of the transformation parameter errors can be calculated using the following formula:

(13) $$U^{2}(q)=C^{+}U^{2}(e){\left(C^{+}\right)}^{T}$$

where *U*(*q*) is a 6 × 6 uncertainty matrix of the transformation parameter error vector *q*, *U*(*e*) is a 3*N* × 3*N* uncertainty matrix of the measurement error vector *e*.

### 2.3. Experimental Verification

**Figure 2:**
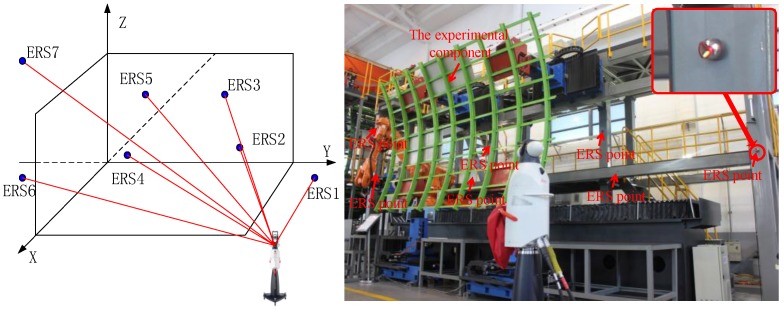
The overall layout of the experiment.

**Figure 3:**
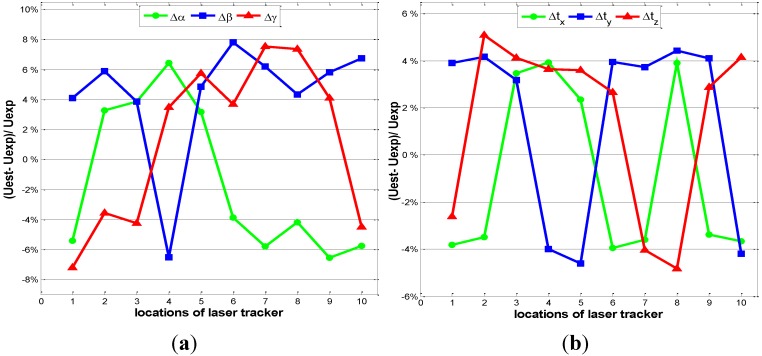
The relative estimation errors of the uncertainties of transformation parameter errors. (**a**) The estimation errors of the uncertainties of rotation parameter errors; (**b**) The estimation errors of the uncertainties of translation parameter errors.

An experiment was implemented to validate the proposed uncertainty estimation model in the factory floor. There are seven ERS points deployed in the fuselage panel assembly system (Figure 2), and one laser tracker (AT-901LR from Leica Company) was used which was well calibrated with full range accuracy of ±15 μm + 6 μm/m. The atmospheric environment in the factory is recorded as follows: the temperature is 22.8 °C, the humidity is 64.3%, and the atmospheric pressure is 974 Pa. In the experiment, the laser tracker was placed at several randomly-selected working locations. In each location, the laser tracker was driven to measure the coordinates of each ERS point 50 times, so the registration process could also be implemented for 50 times. Then, with the obtained 50 groups of the transformation parameters, the variances of transformation parameter errors are calculated and the uncertainties (***U_exp_***) are derived. Finally, the uncertainties ***U_exp_*** are compared with the estimated uncertainties (***U_est_***) calculated with Equation (12). Figure 3 illustrated the relative estimation errors of the transformation parameter errors, in which the maximum estimation error of the uncertainty of the translation parameter error is less than 5%, and the maximum estimation error of the uncertainty of the rotation parameter error is less than 8%. Obviously, the uncertainty estimation model has exhibited considerable performance in the estimation of the uncertainties of the transformation parameter errors.

## 3. Configuration analysis of the ERS points

In aircraft assembly, the configuration of the ERS points is the most important issue in the deployment of the large-volume metrology system. However, the ERS points have been always deployed experientially, and no guidance for the deployment could be followed to improve the measurement uncertainty. With the proposed uncertainty estimation model, the influence of the ERS point configuration that affects the uncertainties of the transformation parameter errors is analyzed in details. Then according to the analysis results, general guidance is established to instruct the deployment of the ERS points.

### 3.1. The Evaluation Criteria

From Equation (13), we can see that the uncertainties of transformation parameter errors are determined by both the configuration matrix and the measurement uncertainties of the ERS points. Expanding Equation (13), the uncertainty of each transformation parameter error is expressed in the following formulas.

(14) $$\begin{array}{l} U^{2}(\Delta \alpha )=\sum_{j=1}^{3N}{\left(C_{1j}^{+}\right)}^{2}U^{2}(e_{j}),U^{2}(\Delta \beta )=\sum_{j=1}^{3N}{\left(C_{2j}^{+}\right)}^{2}U^{2}(e_{j}),U^{2}(\Delta \gamma )=\sum_{j=1}^{3N}{\left(C_{3j}^{+}\right)}^{2}U^{2}(e_{j})\\ U^{2}(\Delta x)=\sum_{j=1}^{3N}{\left(C_{4j}^{+}\right)}^{2}U^{2}(e_{j}),U^{2}(\Delta y)=\sum_{j=1}^{3N}{\left(C_{5j}^{+}\right)}^{2}U^{2}(e_{j}),U^{2}(\Delta z)=\sum_{j=1}^{3N}{\left(C_{6j}^{+}\right)}^{2}U^{2}(e_{j}) \end{array}$$

where *U*(*A*) is the uncertainty of the element *A*, and *C_ij_*^+^ is the component in the *i*th row and *j*th column of matrix ***C^+^***.

Then, let:

(15) $$\begin{array}{l} K_{\alpha }=\sum_{j=1}^{3N}{\left(C_{1j}^{+}\right)}^{2},K_{\beta }=\sum_{j=1}^{3N}{\left(C_{2j}^{+}\right)}^{2},K_{\gamma }=\sum_{j=1}^{3N}{\left(C_{3j}^{+}\right)}^{2}\\ K_{x}=\sum_{j=1}^{3N}{\left(C_{4j}^{+}\right)}^{2},K_{y}=\sum_{j=1}^{3N}{\left(C_{5j}^{+}\right)}^{2},K_{z}=\sum_{j=1}^{3N}{\left(C_{6j}^{+}\right)}^{2} \end{array}$$

**Figure 4:**
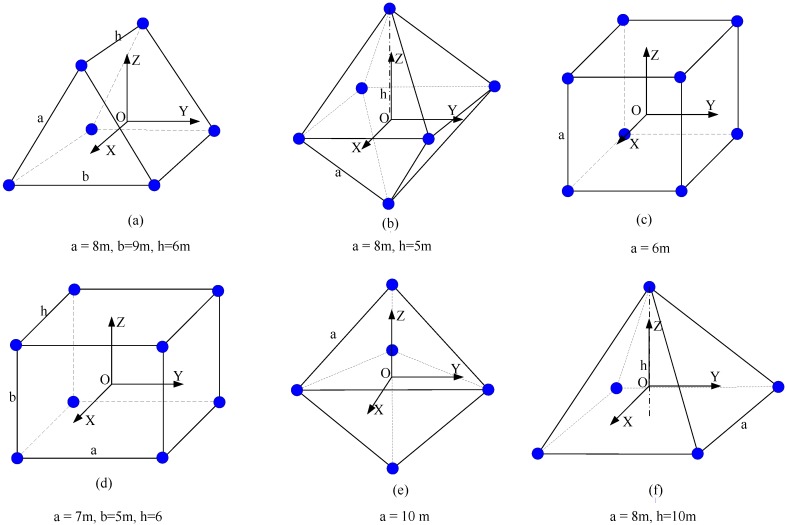
The geometric layouts of the ERS points. (**a**) Triangular prism layout; (**b**) Double rectangular pyramid layout; (**c**) Cube layout; (**d**) Cuboid layout; (**e**) Double triangular pyramid layout; (**f**) Rectangular pyramid layout.

*K_α_*, *K_β_*, *K_γ_*, *K_x_*, *K_y_*, and *K_z_* are named the sensitivity coefficients of the transformation parameter errors, which describes how the uncertainty of transformation parameter error varies with the changes in uncertainties of the ERS points. The values of the sensitivity coefficients are determined only by the configuration matrix ***C*** of the ERS points. When the measurement uncertainties of the ERS points are fixed, the uncertainties of the transformation parameter errors are proportional to the sensitivity coefficients. So, the sensitivity coefficients can be used as criteria to evaluate the quality of the configuration of the ERS points.

The sensitivity coefficients vary with the different configurations of the ERS points. In the following section the influences of the layout of the ERS points and the assembly coordinate system that affect the sensitivity coefficients are analyzed. The mostly used six types of layouts of the ERS points are illuminated in Figure 4. There are six ERS points in layouts (a) and (b), eight ERS points in layouts (c) and (d), and five ERS points in layouts (e) and (f). Each layout has the almost the same size of volume that the ERS points envelop. To simplify the analysis, the laser tracker is placed at the center of gravity of the ERS points in every layout, and the assembly coordinate is supposed to be coincident with the laser tracker’s coordinate system, which are illuminated in Figure 4.

### 3.2. The Impact of the Layout of the ERS Points

The layout of the ERS points is one of the most important configurations, and the sensitivity coefficients of different layouts of the ERS points with the same point number and volume size are various. Table 1 lists the sensitivity coefficients of six different layouts of the ERS points. In layouts (a) and (b), there are equal point numbers and same volume sizes; however, the sensitivity coefficients of the rotation parameter errors are unequal. Moreover, the same situation occurs in layouts (c) and (d) and layout (e) and (f). The layout of the ERS points affects only the rotation parameter errors but not the translation parameter errors. In Table 1, the sensitivity coefficients *K_x_*, *K_y_*, and *K_z_* in each layout are equivalent; this is because the origin of the assembly coordinate system coincides with the center of gravity of the ERS points, which will be discussed in Section 3.3. In layout (c), the sensitivity coefficients of the rotation parameters are also equivalent due to its particular geometric layout.

**Table 1:** The sensitivity coefficients of transformation parameter errors.

Layout	*K_α_*	*K_β_*	*K_γ_*	*K_α_ + K_β_ + K_γ_*	*K_x_*	*K_y_*	*K_z_*	*K_x_ + K_y_ + K_z_*
a	0.0080	0.0069	0.0052	0.0200	0.1667	0.1667	0.1667	0.5000
b	0.0088	0.0088	0.0078	0.0254	0.1667	0.1667	0.1667	0.5000
c	0.0069	0.0069	0.0069	0.0208	0.1250	0.1250	0.1250	0.3750
d	0.0082	0.0059	0.0068	0.0208	0.1250	0.1250	0.1250	0.3750
e	0.0055	0.0055	0.0100	0.0209	0.2000	0.2000	0.2000	0.6000
f	0.0069	0.0069	0.0078	0.0217	0.2000	0.2000	0.2000	0.6000

**Figure 5:**
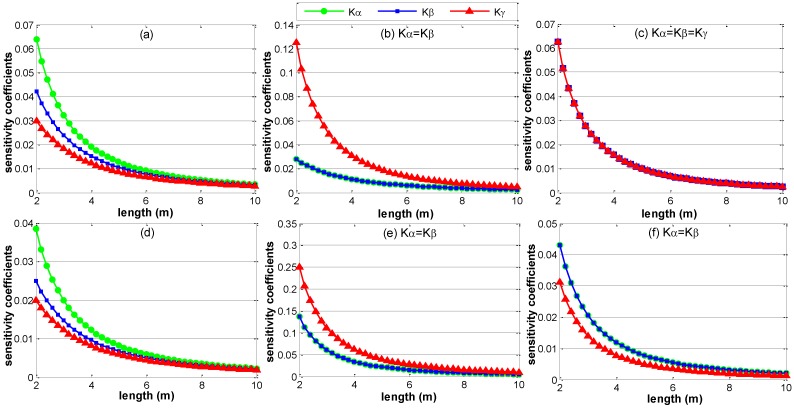
The sensitivity coefficients of rotation parameter errors versus volume size. (**a**) Sensitivity coefficients of layout a; (**b**) Sensitivity coefficients of layout b; (**c**) Sensitivity coefficients of layout c; (**d**) Sensitivity coefficients of layout d; (**e**) Sensitivity coefficients of layout e; (**f**) Sensitivity coefficients of layout f.

In addition to the layout, the sensitivity coefficients are also determined by the size of the volume that the ERS points envelop. For a specific layout of the ERS points, the sensitivity coefficients of rotation parameter errors will decrease when the volume size increases. Figure 5 shows the sensitivity coefficient curves of rotation parameter errors, in which the x-axis denotes the side length “*a*” of the layout in Figure 4. During the expansion of the volume size, the dimension of length “a” increases from 2 m to 10 m with an increase of 0.4 m in each step, and the other dimensions of each layout increases proportionally. From Figure 5, we can see that the sensitivity coefficients of rotation parameter errors decrease very quickly when the side length increases at first, and then the decrease velocity becomes slow when the side length increases unceasingly. Obviously, the configuration of the ERS points with larger volume size has smaller sensitivity coefficients. In layout (c), the sensitivity coefficients of rotation parameters remain equivalent during the expansion of the volume size. The sensitivity coefficients of translation parameter errors remain constant when the volume size is expanded, as the origin of the assembly coordinate system is not moved.

**Figure 6:**
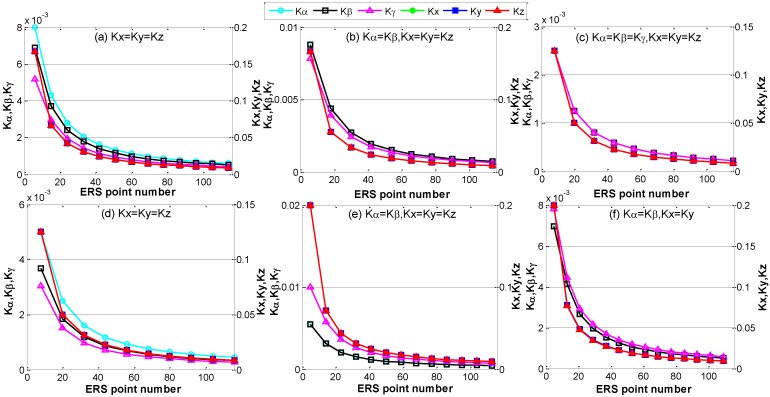
The sensitivity coefficients of transformation parameter errors versus ERS point number. (**a**) Sensitivity coefficients of layout a; (**b**) Sensitivity coefficients of layout b; (**c**) Sensitivity coefficients of layout c; (**d**) Sensitivity coefficients of layout d; (**e**) Sensitivity coefficients of layout e; (**f**) Sensitivity coefficients of layout f.

The sensitivity coefficients are also determined by the number of the ERS points. To retain the geometric layout and volume size, the number of the ERS points is increased by inserting extra points in each geometric side uniformly. When the number of ERS points increases, the sensitivity coefficients of transformation parameter errors are decreased (Figure 6). In Figure 6, the left vertical coordinate denotes the sensitivity coefficients of translation parameter errors, and the right vertical coordinate denotes the sensitivity coefficients of rotation parameter errors. Analogous to the influence of volume size, the sensitivity coefficients of transformation parameter errors reduce very quickly at first, and then the decrease velocity becomes slow when the number of the ERS points increases unceasingly. Obviously, the configuration of ERS points with more point number has smaller sensitivity coefficients.

### 3.3. The Impact of the Assembly Coordinate System

In addition to the layout of the ERS points, the sensitivity coefficients are also dependent on the position and orientation of the assembly coordinate system, as the nominal coordinates of the ERS points are related to the assembly coordinate system. Specifically, the sensitivity coefficients of rotation parameter errors are related to the orientation of the assembly coordinate system, and the sensitivity coefficients of translation parameter errors are related to the position of the assembly coordinate system.

**Figure 7:**
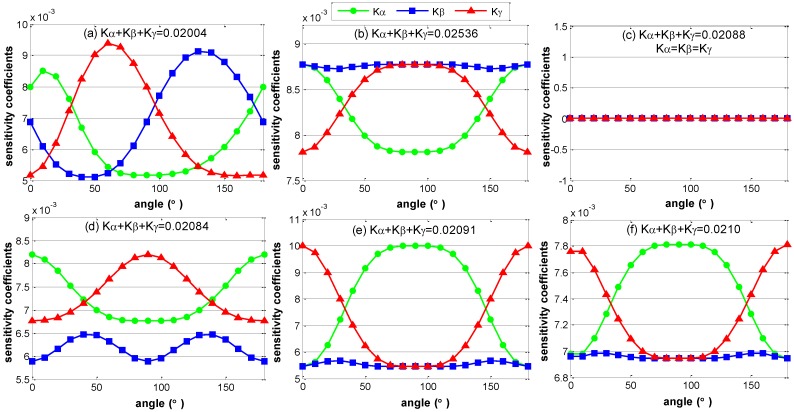
The fluctuation of sensitivity coefficients of rotation parameter errors. (**a**) Sensitivity coefficients of layout a; (**b**) Sensitivity coefficients of layout b; (**c**) Sensitivity coefficients of layout c; (**d**) Sensitivity coefficients of layout d; (**e**) Sensitivity coefficients of layout e; (**f**) Sensitivity coefficients of layout f.

The sensitivity coefficients of translation parameter errors remain constants, and the sensitivity coefficients of rotation parameter errors fluctuate periodically, when the orientation of the assembly coordinates changes. Figure 7 shows the fluctuation of the sensitivity coefficients of rotation parameter errors with the six different layouts, when the coordinate axes of assembly coordinate rotates from 0° to 180°. From Figure 7 we can see that the fluctuations of the sensitivity coefficients are periodic with the period of 180°, and for each layout of the ERS points, the sum of the sensitivity coefficients of rotation parameter errors is a constant. Particularly, the sensitivity coefficients in layout (c) are equivalent and remain constant during the entire period.

The sensitivity coefficients of rotation parameter errors remain unchanged, and the sensitivity coefficients of translation parameter errors vary, when the origin of the assembly coordinate system moves. The sensitivity coefficients of translation parameter errors yield the smallest value when the origin of the assembly coordinate system coincides with the center of gravity of the ERS points and then grow larger when the distance between the origin of the assembly coordinate system and the center of gravity of the ERS points is enlarged. In each layout, the sensitivity coefficients of translation parameter errors are equivalent when the origin of the assembly coordinate system coincides with the center of gravity of the ERS points.

## 4. Conclusions

To optimize the deployment of the ERS points in large-volume metrology system, this paper derived an explicit model to estimate the uncertainties of the transformation parameter errors. Based on the derived model, a group of sensitivity coefficients are proposed to evaluate the quality of the configuration of the ERS points. Then, the influences of the ERS points with several typical configurations that affect the uncertainties of the transformation parameter errors are analyzed using the sensitivity coefficients. Finally general guidance is summarized for deploying the ERS points.
